# In silico identification and molecular characterization of genes predominantly expressed in the fish oocyte

**DOI:** 10.1186/1471-2164-9-499

**Published:** 2008-10-23

**Authors:** Julien Bobe, Thaovi Nguyen, Sophie Mahé, Philippe Monget

**Affiliations:** 1 INRA, UR1337, IFR140, Ouest Genopole, Campus de Beaulieu, F-35000 Rennes, France; 2 INRA, PRC, F-37380, Nouzilly, France

## Abstract

**Background:**

In fish, molecular mechanisms that control follicle-enclosed oocyte progression throughout oogenesis and oocyte developmental competence acquisition remain poorly understood. Existing data in mammals have indicated that the so called "oocyte-specific" genes play an important role in oogenesis, fertilization, and early embryo development. In teleost species, very little is known about "oocyte-specific" genes. The present study therefore aimed at identifying and characterizing oocyte-specific genes in fish.

**Results:**

Using digital differential display PCR, mouse ESTs exhibiting an oocyte-predominant expression were identified. Those murine ESTs were subsequently used to identify cognate rainbow trout (*Oncorhynchus mykiss*) ESTs using a reciprocal Blast search strategy. In the present study we report the identification of five previously uncharacterized rainbow trout cDNAs exhibiting a oocyte-specific, oocyte-predominant, or gonad-specific expression: zygote arrest 1 (*zar1*), v-mos Moloney murine sarcoma viral oncogene-like protein (*mos*), B-cell translocation gene (*btg3*), growth differentiation factor 9 (*gdf9*), and mutS homolog 4 (*msh4*). The orthology relationship of each of these genes with vertebrate counterparts was verified by phylogenetic analysis. Among those five genes, three had never been characterized in any fish species. In addition, we report the oocyte-predominant expression of *btg3 *for the first time in any vertebrate species. Finally, those five genes are present in unfertilized eggs as maternally-inherited mRNAs thus suggesting that they could participate in ovarian folliculogenesis as well as early embryonic development.

**Conclusion:**

The expression patterns of *zar1*, *mos*, *btg3*, *gdf9 *and *msh4 *in rainbow trout and the functions of their orthologs in higher vertebrates strongly suggest that they might play an important role in follicle-enclosed oocyte development, meiosis control and early embryonic development in fish. Future investigations are however required to unravel the participation of those strong candidates in the molecular processes that control folliculogenesis and/or oocyte developmental competence in fish.

## Background

Oocyte developmental competence can be defined as the oocyte ability to be fertilized and to subsequently develop into a normal embryo. In fish, molecular mechanisms that control oocyte developmental competence remain poorly understood. In the past few years, transcriptomic investigations have been initiated to tentatively link oocyte transcriptome and oocyte developmental potential in order to identify key genes involved in the control of oocyte developmental competence [1]. While these types of approaches have been successful, information on the specific molecular mechanisms that make a good oocyte are still limited. One alternative way to fully understand the molecular mechanisms controlling oocyte quality is to study genes that are specifically or predominantly expressed in the oocyte. In mammals it has been shown that the so called "oocyte-specific" genes can affect folliculogenesis, fertilization and early development [2-4]. These genes have been extensively studied in mammals. Yet, very little information is available about those genes in fish despite the recent identification of ovarian-predominant genes in zebrafish [5]. The purpose of the present study was therefore to identify and characterize genes exhibiting a predominant oocyte expression in fish. Taking advantage of the numerous murine tissue-specific libraries available in public databases, we used an *in silico *approach to identify genes exhibiting an oocyte-predominant expression in rainbow trout (*Oncorhynchus mykiss*). Our study led to the identification and characterization of five previously uncharacterized rainbow trout cDNAs exhibiting an oocyte-specific, oocyte-predominant, or gonad-specific expression: zygote arrest 1 (*zar1*), v-mos Moloney murine sarcoma viral oncogene-like protein (*mos*), B-cell translocation gene (*btg3*), growth differentiation factor 9 (*gdf9*), and mutS homolog 4 (*msh4*).

## Results

### Zygote Arrest 1 (zar1)

**Figure 1:**
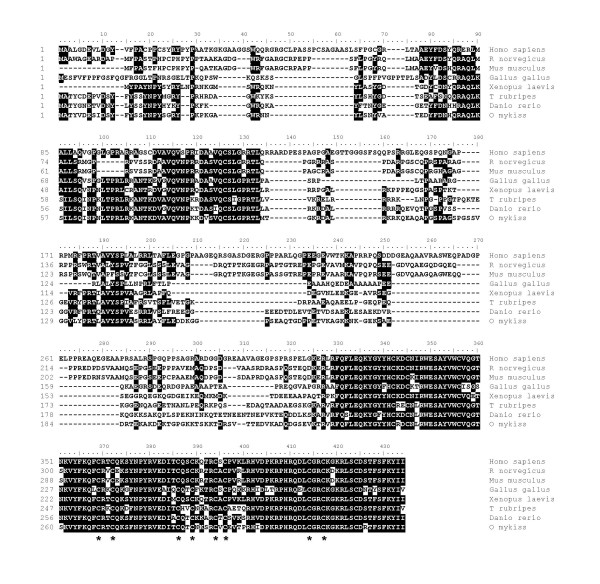
**Alignment of vertebrate ZAR1 amino acid sequences**. Comparison of rainbow trout zar1 amino acid sequence (ABV25059) with human (NP_783318), mouse (NP_777366), rat (EDL89977), chicken (XP_001234452), xenopus (NP_001083958), fugu (NP_001027939), and zebrafish (NP_919362) amino acid sequences. Shaded areas indicate identical amino acids. Asterisks denote the conserved cysteines of the atypical PHD motif.

**Figure 2:**
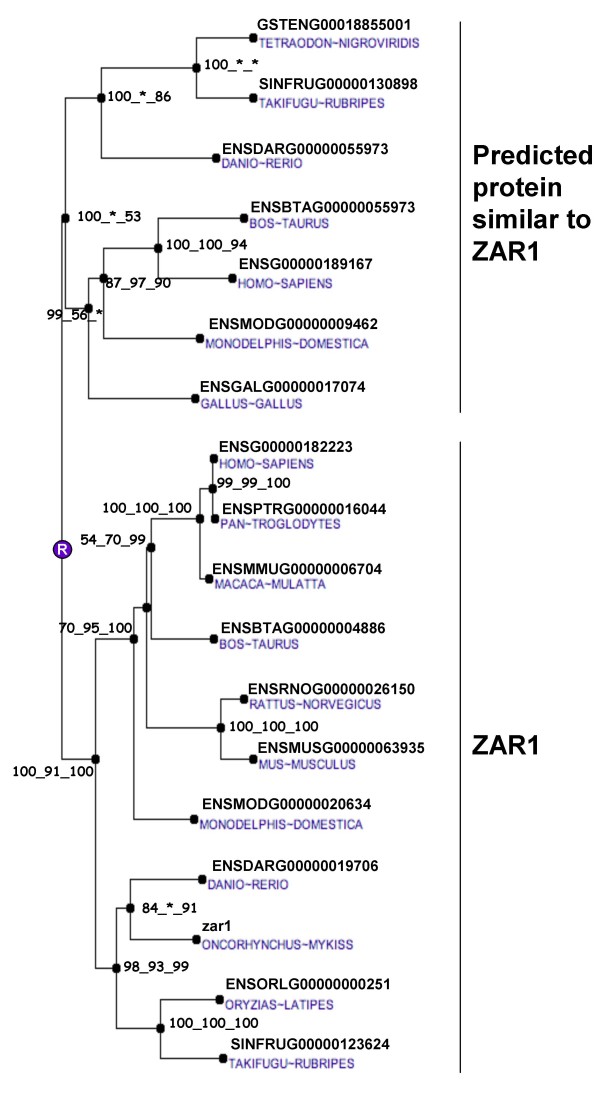
**Phylogenetic tree of ZAR1 proteins**. The phylogenetic tree was built from protein sequences using the Ensembl database. The tree is the fusion on the NJ topology, of three phylogenetic trees built based on neighbour joining, maximum parsimony, and maximum likelihood. For each node, bootstrap values are reported for each npl method. An asterisk indicates that the bootstrap value is lower than 50%. Bootstrapping was carried out with 1000 replications. "R" node represents the ancestral gene.

**Figure 3:**
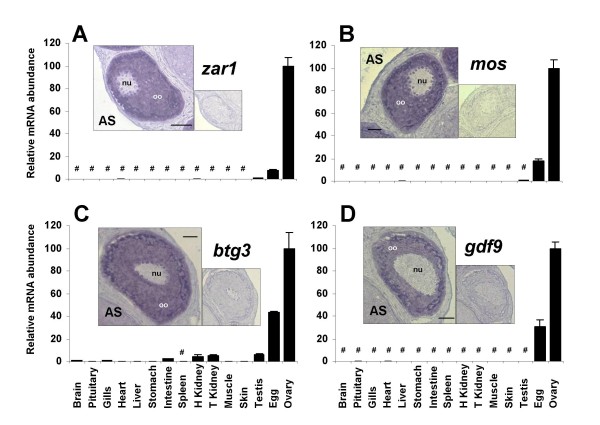
**Tissue expression of rainbow trout *zar1*, *mos*, *btg3 *and *gdf9***. Tissue expression of rainbow trout *zar1, mos, btg3 *and *gdf9 *transcripts. Real-time PCR analysis was conducted using total RNA originating from the following tissues sampled in 3 different fish: brain, pituitary, gills, heart, liver, stomach, intestine, spleen, head kidney, trunk kidney, muscle, skin, ovary, unfertilized eggs (i.e. metaphase II oocytes), and stage II testis. For each tissue, 3 separate reverse transcription reactions were carried out using separate RNA samples originating from 3 different fish. Reverse transcription reactions were pooled and use to run real-time PCR in triplicates. Mean and standard deviation are shown (N = 3). Expression levels not significantly different from background signal at p < 0.05 are indicated (#). Expression levels are expressed as a percentage of the expression in the ovary. In situ hybridization of rainbow trout ovarian tissue sections in the presence of antisense (AS) probe. Labels: **nu **= nucleus, **oo **= ooplasm. Bars represent 200 μm. A smaller view of an adjacent section hybridized with the sense probe is shown for each gene.

The nucleotide sequence of rainbow trout *zar1 *cDNA was 1195 bp in length (EU124662) and presumably encoded for a 333-aa protein. The encoded protein (ABV25059) had an estimated molecular mass of 38 kDa. The rainbow trout zar1 protein exhibited 64%, 60%, and 41% sequence identity with zebrafish (*Danio rerio*), Xenopus and Human zar1 proteins respectively (Figure 1) and the phylogenetic analysis showed that rainbow trout zar1 was orthologous to ZAR1 proteins previously characterized in vertebrates (Figure 2). As previously reported for other species, the zar1 rainbow trout sequence exhibited an atypical Plant Homeo Domain (PHD) finger in its C-terminal region (Figure 1). Real-time PCR data showed that *zar1 *was strongly expressed in the ovary (Figure 3A). The transcript was also present in unfertilized eggs thus demonstrating that trout zar1 is maternally-inherited. Finally, *zar1 *transcript could be detected in testis but not in any other tissue. In the ovary, *in situ *hybridization data showed that *zar1 *was expressed in previtellogenic oocytes (Figure 3A).

### v-mos Moloney murine sarcoma viral oncogene-like protein (mos)

**Figure 4:**
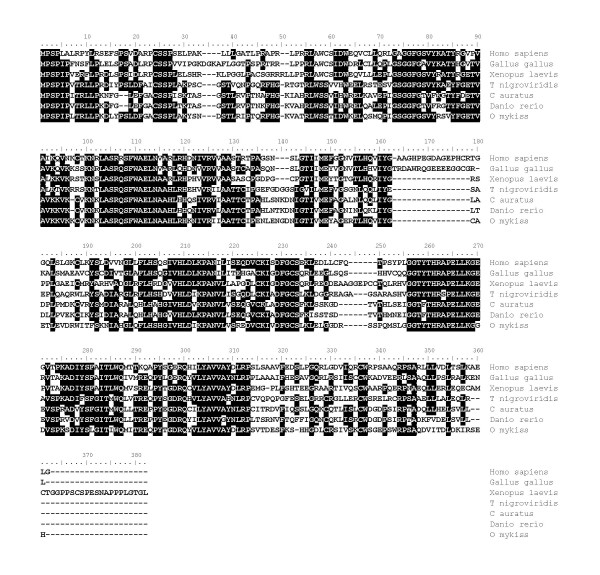
**Alignment of vertebrate MOS amino acid sequences**. Comparison of rainbow trout mos amino acid sequence (ABX64430) with human (NP_005363), chicken (NP_001026687), xenopus (NP_001081563), tetraodon (CAF92637), goldfish (BAA85880) and zebrafish (NP_991143) amino acid sequences.

**Figure 5:**
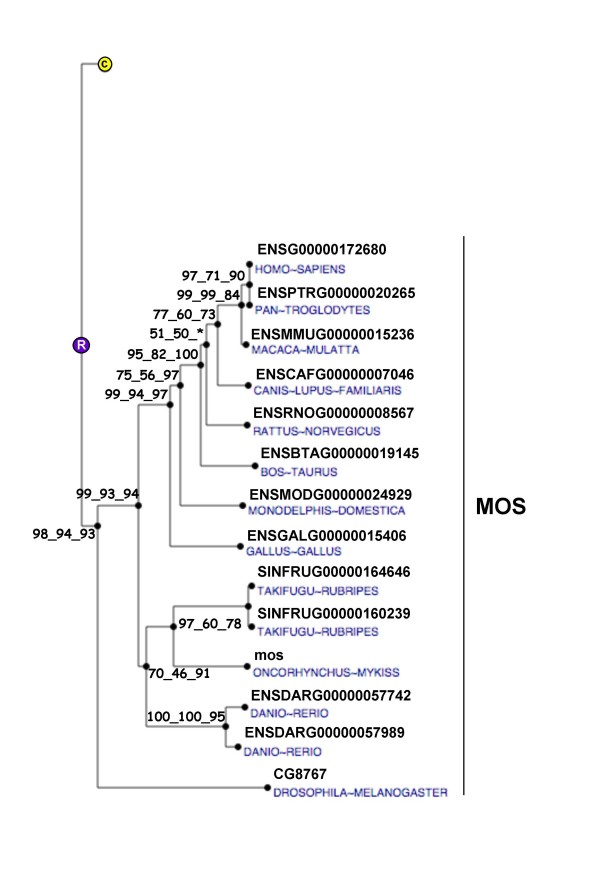
**Phylogenetic tree of MOS proteins**. The phylogenetic tree was built from protein sequences using the Ensembl database. The tree is the fusion on the NJ topology, of three phylogenetic trees built based on neighbour joining, maximum parsimony, and maximum likelihood. For each node, bootstrap values are reported for each npl method. An asterisk indicates that the bootstrap value is lower than 50%. Bootstrapping was carried out with 1000 replications. "R" node represents the ancestral gene. For clarity reasons, the branch corresponding to paralogous genes were reduced and indicated by a circled C.

The nucleotide sequence of rainbow trout *mos *cDNA was 1530 bp in length (EU276588) and presumably encoded for a 335-aa protein. The encoded protein (ABX64430) had an estimated molecular mass of 37 kDa. The rainbow trout mos protein exhibited 62%, 51%, and 48% sequence identity with zebrafish, Xenopus and Human MOS proteins respectively (Figure 4). The phylogenetic analysis showed that rainbow trout mos was orthologous to previously characterized MOS proteins (Figure 5). Real-time PCR data showed that *mos *was strongly expressed in the ovary (Figure 3B). The transcript was also present in unfertilized eggs thus demonstrating that *mos *is maternally-inherited. Finally, *mos *transcript could not be detected in any other tissue. In the ovary, *in situ *hybridization data showed that *mos *was expressed in previtellogenic oocytes (Figure 3B).

### B-cell translocation gene (btg3)

**Figure 6:**
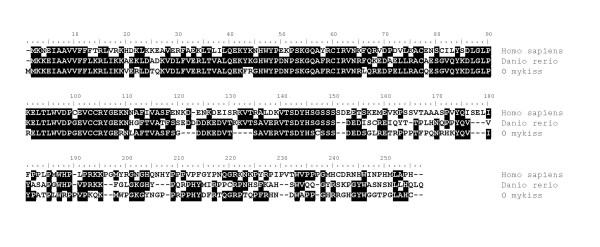
**Alignment of vertebrate BTG3 amino acid sequences**. Comparison of rainbow trout btg3 amino acid sequence (ACE74545) with human (NP_006797), and zebrafish (XP_707837) amino acid sequences.

**Figure 7:**
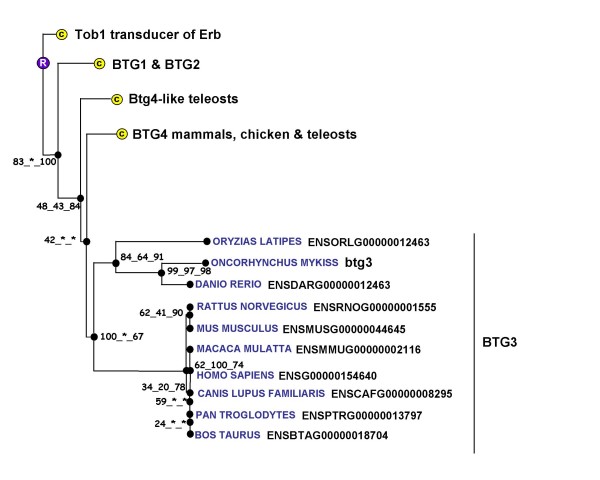
**Phylogenetic tree of BTG3 proteins**. The phylogenetic tree was built from protein sequences using the Ensembl database. The tree is the fusion on the NJ topology, of three phylogenetic trees built based on neighbour joining, maximum parsimony, and maximum likelihood. For each node, bootstrap values are reported for each npl method. An asterisk indicates that the bootstrap value is lower than 50%. Bootstrapping was carried out with 1000 replications. "R" node represents the ancestral gene. For clarity reasons, the branches corresponding to paralogous genes were reduced and indicated by a circled C.

The nucleotide sequence of rainbow trout *btg3 *was 1385 bp in length (EU723246) and presumably encoded for a 237-aa protein. The encoded protein had an estimated molecular mass of 27 kDa. The rainbow trout btg3 protein exhibited 64% and 48% sequence identity with a predicted zebrafish sequence and Human BTG3 protein respectively (Figure 6) and the phylogenic analysis showed that the rainbow trout btg3 was orthologous to BTG3 proteins previously characterized in vertebrates (Figure 7). Real-time PCR data showed that *btg3 *was strongly expressed in the ovary and in unfertilized eggs thus demonstrating that *btg3 *is maternally-inherited (Figure 3C). The transcript was also present at lower levels in testis, kidney, intestine, gills and brain. Finally, *btg3 *transcript could be detected at extremely low levels in several other tissues but not in spleen. In the ovary, *in situ *hybridization data showed that *btg3 *was expressed in the previtellogenic oocyte (Figure 3C).

### Growth differentiation factor 9 (gdf9)

**Figure 8:**
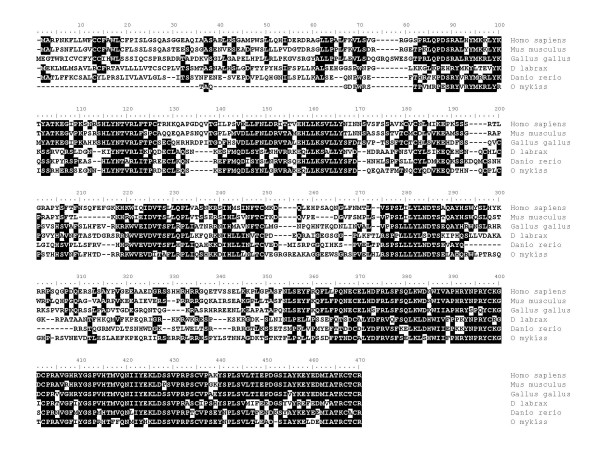
**Alignment of GDF9 amino acid sequences in vertebrates**. Comparison of rainbow trout gdf9 amino acid sequence (ACE74544) with seabass (CAP71884), zebrafish (NP_001012383), chicken (AAT74587), mouse (NP_032136) and human (NP_005251) GDF9 proteins.

**Figure 9:**
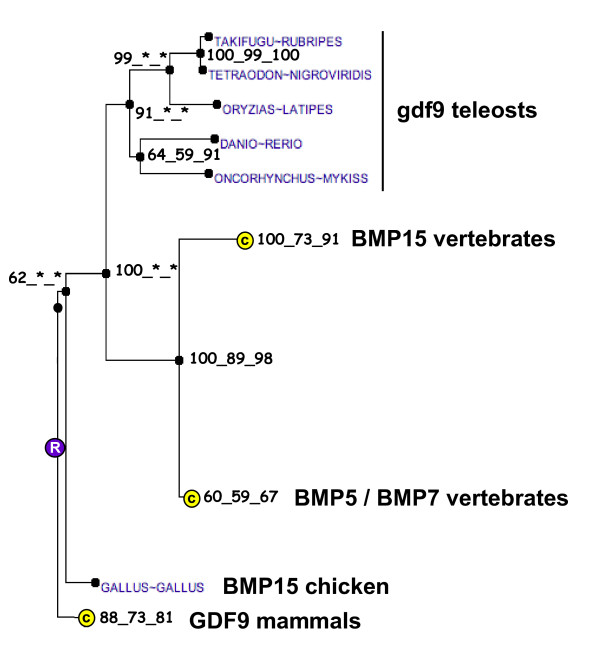
**Phylogenetic tree of GDF9 proteins**. The phylogenetic tree was built from protein sequences using the Ensembl database. The tree is the fusion on the NJ topology, of three phylogenetic trees built based on neighbour joining, maximum parsimony, and maximum likelihood. For each node, bootstrap values are reported for each npl method. An asterisk indicates that the bootstrap value is lower than 50%. Bootstrapping was carried out with 1000 replications. "R" node represents the ancestral gene. For clarity reasons, some of the branches corresponding to paralogous genes or orthologous genes in higher vertebrates were reduced and indicated by a circled C.

For gdf9, a partial rainbow trout cDNA (EU723245) was sequenced. The deduced rainbow trout gdf9 aa sequence exhibited 56%, 56%, 47%, 43% and 40% sequence identity with zebrafish, seabass, chicken, mouse and Human GDF9 proteins respectively (Figure 8) and the phylogenic analysis clearly showed that the rainbow trout gdf9 was orthologous to gdf9 proteins previously characterized in teleosts (Figure 9) [6,7]. In contrast, the topology of the tree vertebrates is not consistent with orthology relationships of GDF9 and BMP15 among vertebrates. This artifact could be due to a long-branch attraction phenomenon. Real-time PCR data showed that *gdf9 *was strongly expressed in the ovary (Figure 3D). The transcript was also present in unfertilized eggs thus demonstrating that *gdf9 *is maternally-inherited. Finally, *gdf9 *transcript could not be detected in any other tissue. In the ovary, *in situ *hybridization data showed that *gdf9 *was expressed in previtellogenic oocytes (Figure 3D).

### mutS homolog 4 (msh4)

**Figure 10:**
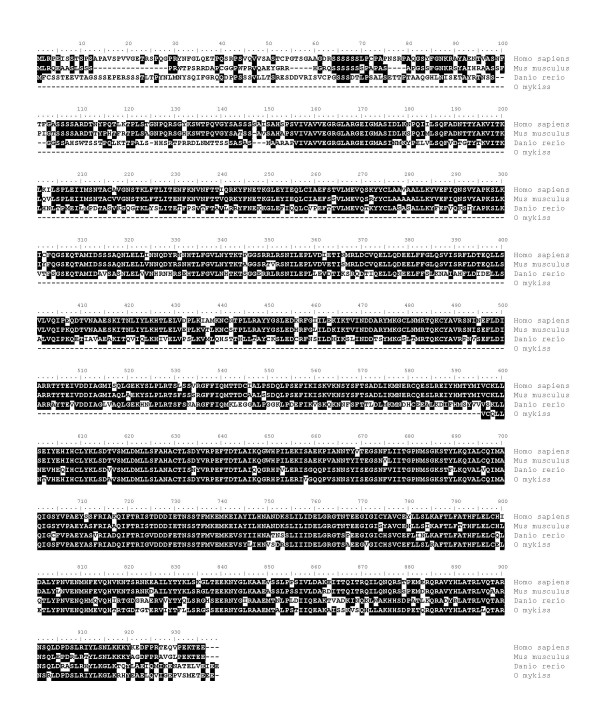
**Alignment of MSH4 amino acid sequences in vertebrates**. Comparison of rainbow trout msh4 amino acid sequence (ACE74546) with zebrafish (XP_688406), mouse (AAL18350), and human (AAB72039) MSH4 proteins.

**Figure 11:**
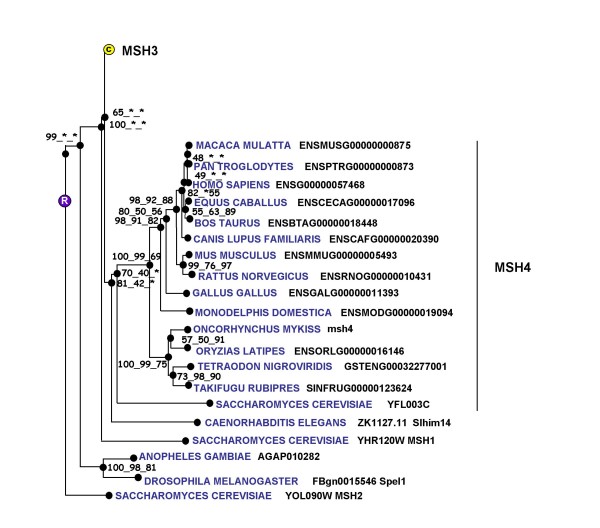
**Phylogenic tree of MSH4 proteins**. The phylogenetic tree was built from protein sequences using the Ensembl database. The tree is the fusion on the NJ topology, of three phylogenetic trees built based on neighbour joining, maximum parsimony, and maximum likelihood. For each node, bootstrap values are reported for each npl method. An asterisk indicates that the bootstrap value is lower than 50%. Bootstrapping was carried out with 1000 replications. "R" node represents the ancestral gene. For clarity reasons, the branch corresponding to MSH3 paralogous genes was reduced and indicated by a circled C.

**Figure 12:**
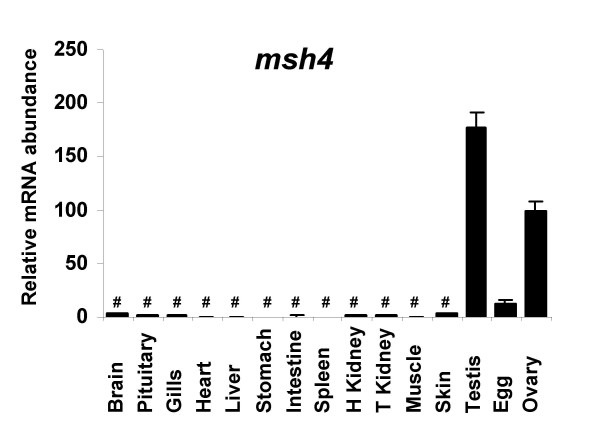
**Tissue expression of rainbow trout *msh4***. Tissue expression of rainbow trout *msh4 *transcript. Real-time PCR analysis was conducted using total RNA originating from the following tissues sampled in 3 different fish: brain, pituitary, gills, heart, liver, stomach, intestine, spleen, head kidney, trunk kidney, muscle, skin, ovary, unfertilized eggs, and stage II testis. For each tissue, 3 separate reverse transcription reactions were carried out using separate RNA samples originating from 3 different fish. Reverse transcription reactions were pooled and use to run real-time PCR in triplicates. Mean and standard deviation are shown (N = 3). Expression levels not significantly different from background signal at p < 0.05 are indicated (#). Expression levels are expressed as a percentage of the expression in the ovary.

For msh4, a partial rainbow trout cDNA (EU723247) was sequenced. The deduced rainbow trout msh4 aa sequence exhibited 66% and 67% sequence identity with mouse and Human MSH4 proteins respectively while it exhibited 81% with an aa sequence deduced from the zebrafish genome (Figure 10) and the phylogenic analysis showed that the rainbow trout msh4 was orthologous to MSH4 proteins previously characterized in vertebrates (Figure 11). Real-time PCR data showed that *msh4 *was strongly expressed in the ovary and in the testis (Figure 12). The transcript was also present at low but detectable levels in unfertilized eggs but could not be detected in any other tissue.

## Discussion

### Zygote Arrest 1 (zar1)

Zygote arrest 1 (Zar 1) is a maternal-effect gene critical for the oocyte-to-embryo transition first identified in the mouse [8]. In this species, zar1^-/- ^mice are infertile as most of their embryos stop developing at one-cell stage. Since its discovery, Zar1 was characterized and its expression studied in several vertebrates species including mammals [9-12], chicken [13] and Xenopus [12]. By contrast, available data in fish are scarce. Zebrafish (*Danio rerio*) and pufferfish (*Fugu rubipres*) *zar1 *sequences have been reported but no information is available on tissue or cellular expression of *zar1 *in any fish species. Similarly to what has been reported in all studied vertebrate species [12], the rainbow trout zar1 sequence exhibits an atypical PHD motif (C-X_2_-C-X_13_-C-X_2_-C-X_4_-C-X_1_-C-X_17_-C-X_2_-C) (Figure 1). The phylogenic analysis confirmed that the rainbow trout zar1 sequence was orthologous to the previously characterized vertebrate ZAR1 sequences including mouse Zar1 (Figure 2). We also show, for the first time in any fish species, that rainbow trout *zar1 *is strongly expressed in the ovary whereas a limited expression is observed in metaphase II oocytes (unfertilized eggs). Within the ovary, the expression was limited to the ooplasm as demonstrated by *in situ *hybridization. A very low signal was also observed in testis whereas no detectable expression was seen in any other tissue. In agreement with the results reported here, *Zar1 *was shown to be expressed exclusively in the oocyte in chicken and mouse [8,12,13]. In contrast, expression in other tissues such as testis [8,11], muscle [12], lung [12], and brain [11] was also observed in various vertebrates species. In bovine, pig and human, the mRNA expression observed in the testis results from an alternative splicing of the *ZAR1 *gene [11]. Together, rainbow trout *zar1 *sequence and tissue expression are consistent with a role in oocyte/embryo development in fish that would be similar to what has been shown in the mouse. Further studies are needed to thoroughly explore any relationship between zar1 expression in the oocyte and the acquisition of oocyte developmental competence.

### v-mos Moloney murine sarcoma viral oncogene-like protein (mos)

In the mouse oocyte, *Mos *encodes for a serine-threonine kinase involved in the maintenance of the meiotic arrest at metaphase II [14-16]. A disruption of *Mos *results in spontaneous parthenogenetic activation of oocytes [14,16]. In Xenopus, mos has also long been implicated in the maintenance of the meiotic arrest [17]. In contrast, data on mos function and expression are extremely limited in fish. In the goldfish (*Carassius auratus*), mos is also involved in the metaphase II arrest but does not participates in oocyte maturation [18]. In the present study, we observed that rainbow trout *mos *mRNA is specifically expressed in the oocyte and not detected in any other tissue. Interestingly, *mos *mRNA is present in the unfertilized egg and is therefore maternally inherited. To the best of our knowledge, no information is available on the tissue distribution of the *mos *mRNA in fish. In several mammalian species, *Mos *mRNA was only found in embryos, ovary and testis [19,20]. In addition, *mos *mRNA was found to be expressed in the shark testis [21]. While it is unknown if rainbow trout *mos *is expressed in the testis at other stages, its expression in the oocyte is consistent with existing data in higher vertebrates. Interestingly, the expression of *mos *mRNA in the unfertilized egg suggests that mos could participate in early development in addition to its well documented role in meiotic arrest.

### B-cell translocation gene (btg3)

BTG3 also named ANA and TOB5 belongs to a family of proteins, the BTG family; know for their anti-poliferative activity. In this family, 6 different proteins have been characterized in vertebrates [22]. The phylogenetic analysis carried out in the present study clearly shows that we have identified the rainbow trout *btg3 *cDNA. The *Btg3 *gene was originally cloned in the mouse [23] and reported to be expressed in several cell lines and in a wide variety of murine and human adult tissues [23,24]. Similarly, porcine *Btg3 *mRNA was detected in most tissues assayed [25]. In the present study, *btg3 *mRNA could be detected in many tissues at very low levels. However, a strong and predominant expression was monitored in the oocyte. Together, our observations are consistent with existing data in mammals. However the oocyte-predominant expression of *BTG3 *was never reported in any vertebrate species and a thorough expression analysis will be necessary in other vertebrate species. Interestingly, several studies have shown that BTG4, another BTG family member, was preferentially expressed in the chicken oocyte [13] and in bovine reproductive tissues [26]. In fish, a recent transcriptomic study also revealed that *btg4 *was predominantly expressed in zebrafish ovarian tissue [5].

In the mouse, the 30 kDa protein encoded by the *Btg3 *gene was cell cycle-dependent and peaked at the end of the G1 phase [23]. Overexpression of the human cognate protein resulted in an impaired serum-induced cell cycle progression from the G0/G1 to S phase in NIH3T3 cells [24]. More recently, in an attempt to study DNA damaged-induced genes, BTG3 was identified as a p53 target exhibiting an antiproliferative activity. Together, the predominant oocyte-expression of rainbow trout btg3 and the antiproliferative activity of the cognate protein in mammals suggest that btg3 could play an important role in oocyte development in fish. In addition, the presence of *btg3 *mRNA in the trout female gamete suggests a role for btg3 during early embryonic development, possibly in response to UV-induced DNA damage.

### Growth differentiation factor 9 (gdf9)

*Gdf9 *is an oocyte-specific gene of the TGF beta superfamily involved in folliculogenesis. It participates in the successful transition from primary to secondary follicles and it was previously shown that *Gdf9*-null mice are sterile [27,28]. In fish, gdf9 was very recently characterized in zebrafish [7] and sea bass (*Dicentrarchus labrax*) [6]. In the present study, the phylogenic analysis clearly showed that rainbow trout gdf9 was orthologous to those previously characterized gdf9 proteins in teleosts [6,7] despite the difficulty to construct a reliable phylogenetic tree among vertebrate species. Northern blot analysis showed an ovarian-specific expression of gdf9 in sea bass [6]. Similarly, semi quantitative PCR showed a gdf9 expression in zebrafish oocyte and testis, and possibly a weak signal in follicular cells [7]. In the present study, we clearly showed using real-time PCR and *in situ *hybridization that rainbow trout *gdf9 *is exclusively expressed in the oocyte. Interestingly, significant levels of *gdf9 *mRNA were detected in the unfertilized egg, thus demonstrating that *gdf9 *is maternally inherited in rainbow trout. This observation is supported by semi-quantitative PCR data in zebrafish showing strong mRNA levels at early blastula stage and sharp decrease during gastrulation [7]. While data on *gdf9 *in fish are scarce, the observed expression patterns are consistent with existing data in mammals. However, the functions of gdf9 in fish, including a possible role during early development, remain currently unknown.

### mutS homolog 4 (msh4)

mutS homolog 4 (MSH4) is a meiosis-specific gene belonging to the DNA mismatch repair (MMR) system. In yeast (*Saccharomyces cerevisiae*) MSH4 is required for reciprocal recombination and proper segregation of homologous chromosomes during meiosis I [29]. In humans, MSH4 protein is only found in testis and ovary [30]. In mice, Msh4 plays an essential role in the control of meiotic recombination and a disruption of this gene leads to male and female sterility due to meiotic failure [31]. In fish, very little is known about msh4. To date, msh4 cDNA and protein sequences were never characterized from any fish species and only sequences automatically predicted from zebrafish and tetraodon genomes are available. In rainbow trout, in agreement with existing data in mammals, the tissue distribution study shows a gonad-specific expression pattern and a strong testicular expression. In addition, high expression levels were found in the late vitellogenic ovary, immediately prior to meiosis resumption. Based on existing data in yeast and mammals, it can be speculated that msh4 plays an important role in meiosis in fish. However, the msh4 mRNA is also detected in metaphase II oocytes at low levels. This indicates that *msh4 *mRNA is maternally inherited and could thus participate in early development, possibly through DNA mismatch repair functions. Further investigations are needed to study the expression of msh4 in fish and characterize its participation in oocyte and embryo development.

## Conclusion

Using an in silico analysis, we have successfully identified 5 previously uncharacterized rainbow trout cDNAs exhibiting an oocyte-specific, gonad-specific, or oocyte-predominant expression. Among those 5 genes, 3 had never been characterized in any fish species. In addition, we report the oocyte-predominant expression of btg3 for the first time in any vertebrate species. Finally, expression patterns of those 5 genes in fish and the functions of their orthologs in higher vertebrates strongly suggest that they might play an important role in fish oocyte development, meiotic arrest and early embryonic development.

## Methods

### In silico identification of candidate genes specifically expressed in the oocyte

A differential digital display (DDD) analysis was previously performed with mouse ESTs providing a list of murine oocyte-specific genes [32]. Cognate rainbow trout expressed sequence tags (ESTs) were subsequently identified using a reciprocal blast search strategy. A tblastX search was performed against all rainbow trout expressed sequence tags (ESTs) available in dbEST [33] using oocyte-specific mouse sequences identified *in silico*. The corresponding clones were obtained from INRA-Agenae program resource center (Jouy-en-Josas, France) [34] and fully sequenced in both directions using the dye-termination method (ABI PRISM 310, PE Biosystems). The deduced amino acid sequence was used for sequence alignment and phylogenetic analysis. Alternatively, the amino acid sequence was deduced from rainbow trout ESTs belonging to the same UniGene cluster.

### Phylogenetic analysis

Phylogenetic analysis was performed using the phylogenomic analysis pipeline available in the FIGENIX platform [35]. FIGENIX retrieved sequences, provided multiple sequence alignments, performed phylogenetic reconstruction, and deduced orthology and paralogy relationships (for a detailed description of pipelines and models used, see [35]). For each studied gene, the protein sequence was entered in the phylogenomic inference task, which was run with the default parameters and with Ensembl database (release 49) [36]. We chose the NJ (neighbor joining) topology for the graphical representation. The trees (npl) are the fusion of three phylogenetic trees built based on neighbor joining [37], maximum parsimony, and maximum likelihood [38]. The Dayhoff PAM matrix provided the distance matrix for the NJ method. The evolutionary distance separating sequences is defined as the number of mutational events per site underlying the evolutionary history separating sequences. Thus, evolutionary relations among sequences are represented by the tree structure, where branch length represents the evolutionary distance [38,39]. Thus, evolutionary relations among sequences are represented by the tree structure, where branch length represents the evolutionary distance [38,39]. In phylogenetic tree, bootstrap values are reported on each node for each npl method. Bootstrapping was carried out with 1000 replications.

### Tissue collection and RNA extraction

Investigations were conducted according to the guiding principles for the use and care of laboratory animals and in compliance with French and European regulations on animal welfare. Rainbow trout (*Oncorhynchus mykiss*) in their first reproductive season were obtained from an experimental fish farm (PEIMA, Sizun, France). Fish were deeply anaesthetized in 2-phenoxyethanol (10 mg/ml of water), killed by a blow on the head and bled by gill arch section. Tissues were sampled from 3 ovulated females. Testis samples were obtained from 3 different males at stage II of spermatogenesis [40]. For RNA extraction, tissues were homogenized in Tri-reagent (Molecular Research Center, Cincinnati, OH) at a ratio of 100 mg per ml of reagent and total RNA was extracted according to manufacturer's instruction. For in situ hybridization, ovarian tissue was sampled from an ovulated female, fixed in Dietrick's fixative (10% Formaldehyde, 28. 5% ethanol, 2% glacial acetic acid) at 4°C overnight, rinsed in tap water for 1 hour and held in 50% ethanol until further processing.

### Real-time PCR

Real-time PCR was performed using an I-Cycler IQ (Biorad, Hercules, CA) as previously described [41]. Reverse transcription products were diluted to 1/50 and 5 μl were used for each real-time PCR reaction. Triplicates were run for each RT product. Real-time PCR was performed using a real-time PCR kit provided with a SYBR Green fluorophore (Eurogentec, Belgium) according to the manufacturer's instructions and using 600 nM of each primer (Table 1). After a 2 min incubation step at 50°C and a 10 min incubation step at 95°C, the amplification was performed using the following cycle: 95°C, 20 sec; 62°C, 1 min, 40 times. The relative abundance of target cDNA within sample set was calculated from a serially diluted ovarian cDNA pool using the I-Cycler IQ software. Subsequently, real-time PCR data were normalized using 18S transcript abundance. After amplification, a fusion curve was obtained in order to ensure that a single PCR product had been generated using the following protocol: 10 sec holding followed by a 0.5°C increase, repeated 80 times and starting at 55°C.

**Table 1:** Real-time PCR primers

Name	Acc #	Forward primer	Reverse primer
*zar1*	EU124662	GAACGAGCAAGGTCTACTTCAAG	GCAAGTCTGGCAGGTGATGTC
*mos*	EU276588	GGCGACAGGCAATATGTTTT	CACTTGGACACAATGGATCG
*gdf9*	EU723245	ACGAGCGACTGTGCTTTGTAC	AATGATCCAATGGCTCAGTT
*btg3*	EU723246	AGAGGAGGTGTGCTGCAGAT	CGTCTGAGGAGGAACAGGAG
*msh4*	EU723247	TCTGTCTGCGAATTCCTCCT	ACCTCCATGTGCTGGTTTTC
*18S*		CGGAGGTTCGAAGACGATCA	TCGCTAGTTGGCATCGTTTAT

For all studied genes the GenBank accession number of the rainbow trout cDNA and the sequence of the primers used for the real-time PCR study are shown

### In situ hybridization

Dehydration (increasing ethanol: 15 min in 50% ethanol, twice 15 min in 70% ethanol, 15 min in 80% ethanol, 30 min in 96% ethanol, and 30 min in 96% ethanol/butanol vol/vol), clearing (butanol once for 30 min, and twice for 3 h each), and paraffin infiltration (once for 1 h and twice for 2 h, at 60°C) were performed in a Citadel 1000 tissue processor (Shandon, Pittsburgh, PA). Dehydrated tissues were embedded in plastic molds in paraffin using a HistoEmbedder (TBS88, Medite, Germany).

Digoxigenin-labeled anti-sense RNA probes were produced using the Promega T3/T7 RNA polymerase Riboprobe Combination System as recommended by the manufacturer, using as DNA template a PCR product obtained following amplification of the plasmid inserts with M13 reverse and M13 forward primers. Digoxigenin-labeled riboprobes were then purified by precipitation in ammonium acetate 7.5 M/ethanol for 2 hours at -20°C, and RNA concentrations were measured using a NanoDrop^® ^spectrophotometer. Serial cross-sections of 5 μm were deparaffinized, re-hydrated in TBS (50 mM Tris, pH 7.4, 150 mM NaCl) and post-fixed in 4% PFA for 20 min. ISH was performed using the "In situ Pro, Intavis AG robotic station". Incubation volumes for all ISH steps were set to 250 μl. Digestion was carried out for 20 min with 3 μg/ml of proteinase K. Pre-hybridization (2 h, 60°C) and hybridization (12 h, 60°C) were carried out in 50% formamide, 2 × SSC, 1 × Denhardt, 10% dextran sulfate, and 250 μg/ml tRNA. For hybridization the digoxigenin-labeled anti-sense RNA probes were diluted in hybridization buffer at a final concentration of 3 ng/μl. Washing steps (2 × SSC, 60 min) were performed at 60°C followed by an RNAse treatment at 37°C. The digoxigenin signal was then revealed with an anti-digoxigenin antibody conjugated with alkaline phosphatase (Roche Diagnostics Corp.) and a NBT/BCIP revelation system (Roche Diagnostics Corp.) as recommended by the manufacturer. Slides were mounted with mowiol 4–88 (Calbiochem).

## Authors' contributions

TN performed RNA extraction, quantitative PCR analysis and sequencing and participated in the writing of the manuscript. SM carried out the in situ hybridization analysis and participated in the writing of the manuscript. PM carried out the phylogenetic analyzes and participated in the design of the study and the writing of the manuscript. JB coordinated the study, carried out the gene sequence analysis and drafted the manuscript. All authors read and approved the manuscript.
